# Atypical lymphoid proliferation of the orbit

**DOI:** 10.3205/oc000193

**Published:** 2022-02-25

**Authors:** Lee Tomas Obias Tan, Felice Katrina C. Trio-Ranche

**Affiliations:** 1 University of the Philippines – Philippine General Hospital, Department of Ophthalmology and Visual Sciences, Manila, Philippines

**Keywords:** lymphoproliferative disease, orbital mass, atypical lymphproliferative disease

## Abstract

**Objective:** Lymphoproliferative disorders are a group of lesions characterized by abnormal proliferation of lymphocytes. In the orbit, they can occur in the ocular adnexae. These neoplasms have defined clinical and pathologic characteristics and account for more than 20% of all orbital tumors. Several types of lymphoproliferative lesions have been described in the orbit. One example is lymphoid hyperplasia, which commonly involves the lacrimal gland. A benign lesion like lymphoid hyperplasia will show a general normal archetype of the tissues-involved lacrimal gland. We expect a polyclonal group of cells with more or less normal architecture of a follicle. On the other hand, lymphoma will show less organized arrangement of cells, and we expect them to be of monoclonal lineage.

**Methods:** This is a case report of a 55-year-old Filipino female who came in for blurring of vision of both eyes. During her assessment, there was an incidental finding of bilateral upper eyelid swelling, and a 30x15 mm palpable firm mass under the right superior orbital rim and a 30x10 mm mass under the left were noted. The right globe was displaced inferiorly, but no proptosis was seen on exophthalmometry. On plain CT scan, we noted a homogenous mass with molding or contouring around the orbital structures. On coronal view, we noted homogenous masses that mold around the globe and recti, and this also confirmed on axial cuts. A section biopsy was done via anterior orbitotomy.

**Results:** Our patient’s histopath had features of both. On scanning magnification, we noted a very cellular round cell tumor. The round cell lesion seemed to be reminiscent of a germinal center of a lymph node. Around it we noticed the glandular structures, which were expected, since this specimen was from the lacrimal gland. A closer view of the lesion on high power showed these lymphocytes within a germinal center. These are large macrophages that actively phagocytose apoptotic lymphoid cells in germinal centers. We expect to see a lot of them in benign hyperplasia, but only a few will be present in malignancies. In summary, our patient had both benign and malignant features, resulting in a histopath result of atypical lymphoid proliferation.

**Conclusion:** Atypical lymphoid proliferation is a rare orbital tumor with benign and malignant features. There is no standard protocol for treatment, and proper multi-specialty coordination is important. External beam radiation therapy with linear accelerator (LINAC) appears to be an effective treatment, with no recurrence in our patient after 5 months.

## Introduction

Lymphoproliferative disorders are a group of lesions characterized by abnormal proliferation of lymphocytes. In the orbit, they can occur in the ocular adnexae. These neoplasms have defined clinical and pathologic characteristics and account for more than 20% of all orbital tumors. Several types of lymphoproliferative lesions have been described in the orbit. One example is lymphoid hyperplasia, which commonly involves the lacrimal gland. In the literature, this is sometimes termed ‘reactive lymphoid hyperplasia’ or ‘benign lymphoid hyperplasia’, because it is characterized by a polyclonal lymphocytic infiltrate with no markers for malignancy [1].

In recent decades, it has been discovered that these lesions occupy a spectrum, with lymphoid hyperplasia on the benign end, and orbital lymphoma on the malignant end. These lesions have the same clinical presentation and the same radiologic features. They only differ histologically. However, there are rare lesions that show both benign and malignant features on histopath. They occupy the middle of the spectrum and are called ‘atypical lymphoid hyperplasia’ [1].

The objectives of this case report were to present a case of atypical lymphoid proliferation of the orbit, and to discuss the management options currently available.

## Case description

Our patient is a 55- year-old female from Quezon province, who first consulted in the previous year for blurring of vision of both eyes. The patient experienced gradually progressive blurring of vision starting 2 years prior to the consultation. She had no other ocular symptoms. One year ago, she was seen at the general clinic, where she was noted to have bilateral cataract and increased cup-disc ratio. She was then referred to the glaucoma service. On follow-up at the glaucoma clinic, the colleagues noted an incidental finding of bilateral eyelid swelling, and referred the patient to the orbit service. Until this point, the patient had not noticed that her upper lids were unusually swollen.

The patient has a family history of hypertension and endometrial CA. The rest of the history is noncontributory.

On physical examination at the orbit clinic, the patient’s best corrected visual acuity was 20/40 of both eyes. Her pupils were 3mm with no afferent pupillary defect. The patient had nuclear sclerosis on both eyes, and slightly enlarged cup disc ratios, 0.5 on the right and 0.6 on the left. Extraocular muscles and tonometry were normal.

**Figure 1:**
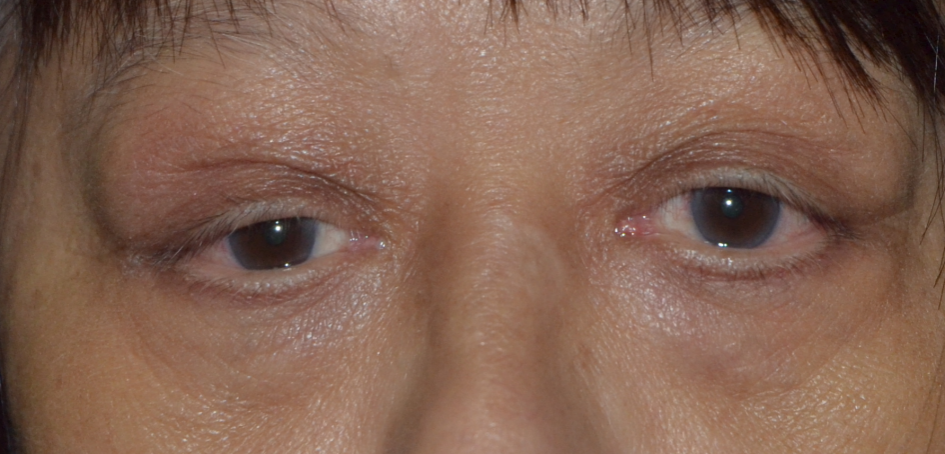
Gross picture of the patient with orbital lymphoproliferative disease

Grossly, there was bilateral upper lid swelling with a 30x15 mm palpable firm mass under the right superior orbital rim, and a 30x10 mm mass under the left. The right globe was displaced inferiorly, but no proptosis was seen on exophthalmometry. The right upper eyelid was moderately ptotic with good levator function (Figure 1 (Fig. 1)).

### Differential diagnosis

On the basis of our history and physical exam we can try to narrow down the possible pathology present. To summarize the salient points, we have an adult patient with slow-growing painless masses in the superolateral orbit. Because this is the location of the fossa of the lacrimal gland, one differential diagnosis would be a pleomorphic adenoma.

Also called a benign mixed tumor, this is a relatively common epithelial neoplasm of the lacrimal gland, found mostly in women in their 30s or 40s. Our next differential diagnosis would be an orbital lymphoproliferative disease, which can be a benign or malignant lymphocytic infiltration of the lacrimal gland and surrounding tissues. Considering the patient’s age, which is 55 years, we will lean more towards malignancy.

Our third would be a cavernous hemangioma, an encapsulated vascular tumor, which is the most common orbital neoplasm in adults. Although this is most commonly located in the intraconal space, it can occur anywhere in the orbit. Our fourth differential diagnosis would be a neurilemmoma or schwannoma, another relatively common well-delineated tumor of the orbit that arises from Schwann cells of peripheral nerve sheaths. These are commonly found in the superior orbit.

Orbital masses in general occur unilaterally. Among these 4 differential diagnoses, only one entity can present bilaterally, i.e. orbital lymphoproliferative disorder.

Our assessment at this point was an orbital lymphoproliferative disorder, bilateral; probably lymphoma with mechanical ptosis with good levator function, right upper eyelid; and suspicion of glaucoma in both eyes. To support our assessment, we needed imaging studies, which had fortunately already been ordered previously by the glaucoma specialist. On plain CT scan, we noted a homogenous mass with molding or contouring around the orbital structures.

**Figure 2:**
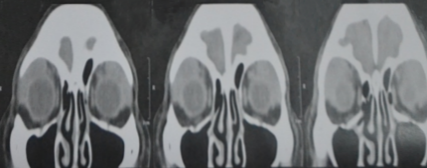
A coronal view of a plain orbital CT scan of the patient showing a homogenous mass molding around the globe and recti bilaterally

**Figure 3:**
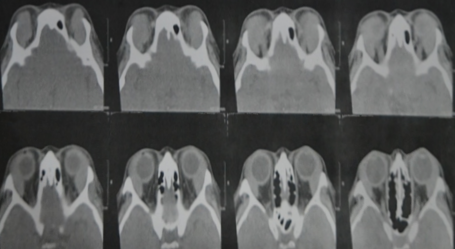
An axial view of a plain orbital CT scan of the patient showing a homogenous mass molding around the globe and recti bilaterally

On coronal view, we noted homogenous masses that mold around the globe and recti, and this also confirmed on axial cuts. This not only supported our assessment, it also effectively ruled out the other 3 differential diagnoses we had earlier (Figure 2 (Fig. 2), Figure 3 (Fig. 3)).

Molding around orbital structures is the radiologic hallmark of orbital lymphoproliferative disorders, but it will not differentiate between benign and malignant disease. Consequently, our assessment was maintained.

**Figure 4:**
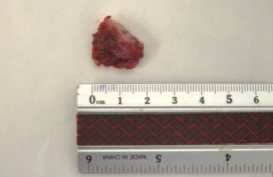
The 20x20 mm square shaped solid grayish mass taken after an incision biopsy was performed via an orbitotomy

Since the only way to differentiate benign vs malignant is on histopath, our new plan was a section biopsy. The approach was via an anterior orbitotomy because the mass was already palpable beneath the superior rim. For our surgery, after subcutaneous anesthesia was injected into the lid, the orbit was approached via a lid crease incision. Dissection up to the superior rim was done in the preseptal plane, and the periosteum was incised to access the orbit. The surgeon obtained a 20x20 mm square-shaped solid grayish mass, which was sent for histopathologic assessment (Figure 4 (Fig. 4)).

### Histopathologic discussion

**Figure 5:**
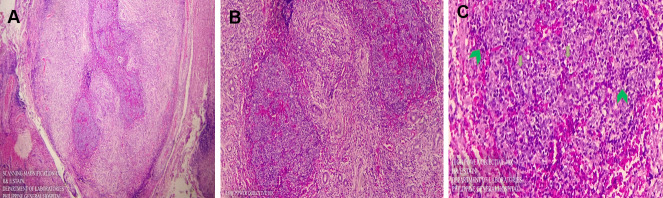
A) On scanning magnification, we noted a very cellular round cell tumor. B) On low objective magnification, the round cell lesion seemed to be reminiscent of a germinal center of a lymph node. C) High power magnification showing lymphocytes within a germinal center.

A benign lesion like lymphoid hyperplasia will show a general normal archetype of the tissues-involved lacrimal gland. We expect a polyclonal group of cells with more or less normal architecture of a follicle. On the other hand, lymphoma will show a less organized arrangement of cells, and we expect them to be of monoclonal lineage. Our patient’s histopathology result had features of both (Figure 5A (Fig. 5)).

On scanning magnification, we noted a very cellular round cell tumor. The round cell lesion seemed to be reminiscent of a germinal center of a lymph node. Around it, we noticed the glandular structures, which were expected, since this specimen was from the lacrimal gland (Figure 5B (Fig. 5)).

A closer view of the lesion on high power showed these lymphocytes (round blue cells on green arrow heads), within a germinal center. Tingible body macrophages could also be seen. These are large macrophages that actively phagocytose apoptotic lymphoid cells in germinal centers. We expect to see a lot of them in benign hyperplasia, but only a few will be present in malignancies (Figure 5C (Fig. 5)).

In summary, our patient had both benign and malignant features, resulting in a preliminary histopathology result of atypical lymphoid proliferation. The plan was then to do immunohistochemical stains for confirmation.

**Figure 6:**
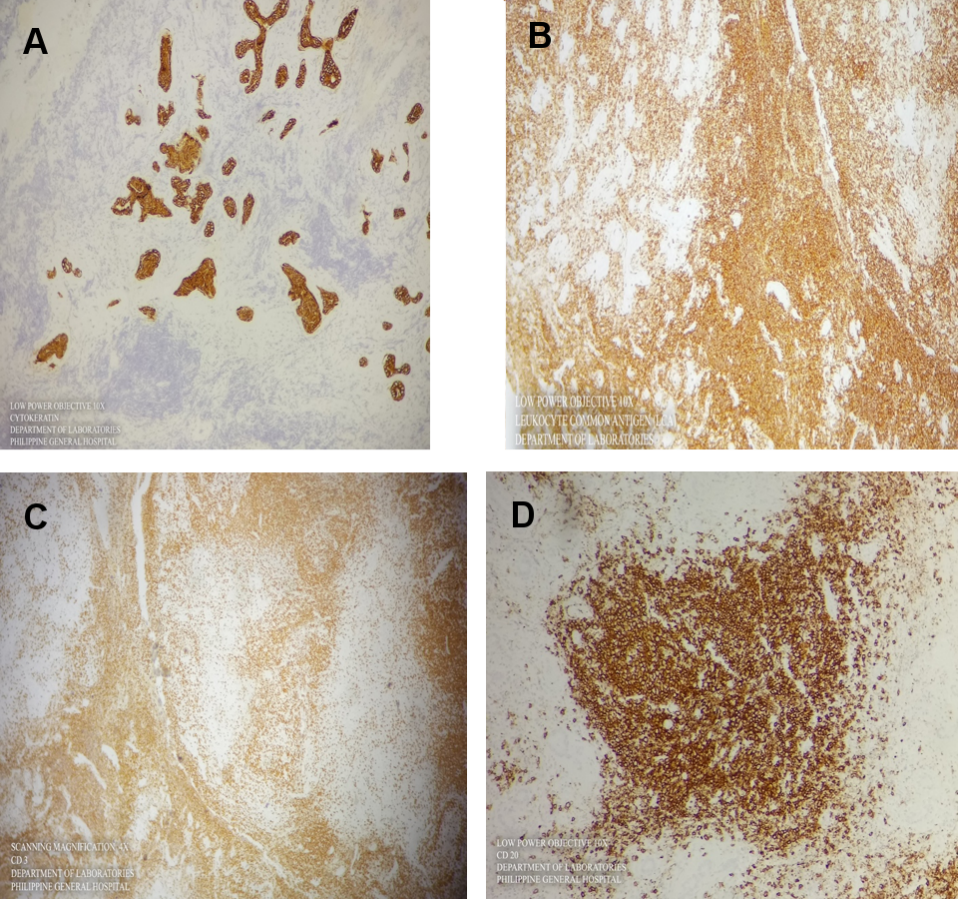
Histochemical staining. A) Cytokeratin was the stain for the epithelial components and here the normal ducts stain positive, while the lesion itself is negative. B) Leukocyte common antigen or CD45 is diffuse and strongly staining, thereby confirming that the lesion is of lymphoid origin. C) CD 3 is a T cell marker and the T cells are usually paracortical in a lymph node. Here, the lymphocytes surrounding the germinal centers light up. D) CD 20 is a B cell marker and they are usually located within the lymphoid follicles. Here the B cells light up with a strong membranous stain.

Four histochemical stains were requested for the patient. The first two will differentiate between epithelial and lymphoid tissue origin. If the specimen stain positive for lymphoid origin, it will then be stained to differentiate between T cell lineage and B cell lineage (Figure 6A (Fig. 6)).

Cytokeratin was the stain for the epithelial components, and here the normal ducts stain positive, while the lesion itself is negative. Leukocyte common antigen or CD45 is diffuse and strongly staining, thereby confirming that the lesion is of lymphoid origin (Figure 6B (Fig. 6)).

CD 3 is a T cell marker, and the T cells are usually paracortical in a lymph node. Here the lymphocytes surrounding the germinal centers light up. CD 20 is a B cell marker, and they are usually located within the lymphoid follicles. The B cells light up with a strong membranous stain (Figure 6C (Fig. 6)).

The information that we have after staining with CD 3 and CD 20 is that the lymphoid lesion is polyclonal, which is a benign feature (Figure 6D (Fig. 6)).

## Discussion

Atypical lymphoid proliferation is a descriptive term used when it is not possible for the pathologist to differentiate between the benign and the malignant nature of a given lymphoid infiltrate. These are biologically indeterminate lesions that have some worrisome clinicopathologic features but cannot be interpreted as malignant lymphomas using all criteria currently available. They have some likelihood for subsequent transformation into lymphomas [2].

A typical lymphoproliferative lesion presents as a gradually progressive, painless mass. These tumors are often located anterior in the orbit or beneath the conjunctiva, where they may show the typical salmon patch appearance.

Lymphoproliferative lesions, whether benign or malignant, usually mold to surrounding orbital structures rather than invading them. Consequently, disturbances of extraocular motility or visual function are unusual. Reactive lymphoid hyperplasias and low-grade lymphomas have a history of slow expansion over a period of months to years [3].

Initial evaluation should start with a thorough history. Like all lymphoproliferative disorders, these present with a gradually progressive proptosis or painless mass. This should be followed by careful physical examination and imaging. Molding around the orbital structures is pathognomonic [3].

For all lymphoproliferative lesions, an open biopsy is preferred. This is to retrieve adequate tissue specimen to establish a diagnosis. Fine needle aspiration will compromise the tissue architecture. Several studies have been done on both ends of the lymphoproliferative spectrum, but no definite treatment has been established for atypical lesions yet [3].

A study by Polito et al. [4] discussed the 3 different treatment options given to patients with atypical lymphoid proliferation. Of the 8 patients with atypical lymphoid proliferation of the orbit without medical or radiotherapy, 37% of the patients progressed to orbital lymphoma. Rituximab is the next option; this is a monoclonal antibody that destroys normal and malignant B cells that have CD 20. It is given through intravenous infusion. The first dose is given slowly over 4 to 5 hours. The rest of the session can be given over a faster period of time, depending on the dose computed by the oncologist on board [4].

Rituximab, a chimeric monoclonal antibody directed against B-cell-specific CD20, has recently gained popularity in the treatment of non-Hodgkin B-cell lymphoma [5]. Srirarm [6] evaluated the therapeutic efficacy of rituximab in 10 patients with lymphoma of which 4 had orbital involvement. Overall, a complete response was achieved in 36% of the patients and the remainder required adjuvant radiotherapy for recurrence. In the presence of systemic involvement, it plays a major role [6].

The last option is radiotherapy, which uses ionizing radiation to kill cancerous cells. A beam or several beams of high energy X-rays are delivered to a patient’s tumor [7]. Most radio-oncologists use the linear accelerator (LINAC). The machine is able to deliver a focused beam of radiation to spare the normal surrounding tissue. Compared to the older methods of radiotherapy, no radioactive sources are placed in the body. External beam radiotherapy alone as a treatment modality has shown a favorable outcome in orbital lymphoma [3].

For treatment planning, the dosimetrist, medical physicist, and radiation oncologist use a special computer program to calculate the radiation dose that will be delivered to the patient’s tumor and the surrounding normal tissue. The radiation oncologist will determine the volume of the tumor and other areas that need to be treated, and outline those on the treatment planning images. He or she will also outline normal structures that should be avoided or considered in devising the treatment plan [3].

In a retrospective study, Polito et al. [4] evaluated 33 cases of lymphoid hyperplasia with a range of 2 to 13 years after radiotherapy, systemic steroid, and no treatment. Of those given radiotherapy, none transformed into non-Hodgkin lymphoma. Among the patients treated with radiation, 71% had a complete response, and 29% had a partial response. Because of the high risk of transformation to non-Hodgkin lymphoma, systemic staging and follow-up is mandatory in all orbital lymphoid tumors [8]. The advised management is radiation therapy [4].

Regarding the treatment options for atypical lymphoid proliferation, there is a stark difference between the prices of using rituximab or external beam radiation therapy. The entire procedure of radiotherapy is covered by Philippine Health Insurance. Although each have their own pros and cons, external beam radiotherapy is the most cost-effective choice for treatment (Table 1 (Tab. 1)).

Because there is no definite protocol, and the possibility of undertreatment and overtreatment have their own risks and benefits, a multidisciplinary conference (MDC) was held to discuss amongst the concerned services what the best course of action would be. Present during the MDC were the radio-oncology service, the medical oncology service, the orbit service, the pathology service, and the patient.

The consensus from the MDC was to do localized radiotherapy. This was then discussed with the patient, including the corresponding side effects. Certain precautions should be taken before starting radiotherapy. The most common ocular side effects of radiotherapy are cataract and dry eye. The patient should be advised of the probability of cataract formation and started on lubricants.

Before treatment, the CT scan of the chest and abdomen were done to rule out any other parts of the body that may have been affected by a similar disease. The patient underwent 10 sessions of external beam radiation therapy at the Cancer Institute from September 26 to October 9, 2017.

The patient was given radiation of 20 Gy (gray) total. The gray is the unit of measurement for the amount of radiation given to the patient.

**Figure 7:**
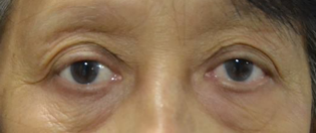
The patient 8 months post radiation therapy with no palpable masses and resolved ptosis

The patient has been on regular follow-up with radio-oncology and our service. The patient had no subjective ocular complaints with no palpable masses and resolution of ptosis. However, BCVA on the left decreased by one line, and the patient was noted to have an anterior subcapsular cataract. A repeat CT scan is not needed according to radiology, but may be done to quantitatively measure the decrease in mass size (Figure 7 (Fig. 7)).

After radiotherapy is done, it is necessary for us to properly follow up our patient.

In a retrospective study by Knowles et al. [9] with a sample size of 108 patients, the patients were treated with the following treatments options: incisional, excisional biopsy, local irradiation, or chemotherapy. Through time, the earliest transformation to lymphoma occurred at 6 months, and the latest was at 82 months.

We plan to have the patient follow up every 6 months.

## Conclusions

Atypical lymphoid proliferation is a rare orbital tumor with benign and malignant features. There is no standard protocol for treatment, and proper multi-specialty coordination is important. External beam radiation therapy with LINAC appears to be an effective treatment, with no recurrence in our patient after 5 months.

## Notes

### Informed consent

Informed consent has been obtained from the patient for the publication of this case report.

### Competing interests

The authors declare that they have no competing interests.

## Figures and Tables

**Table 1:**
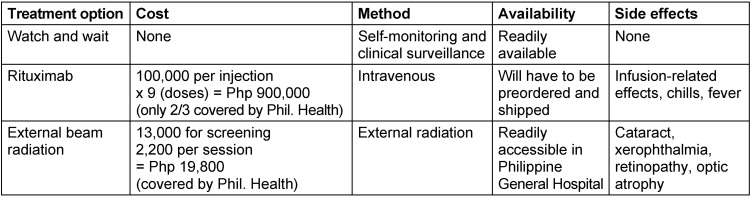
Three treatment options for atypical lymphoid proliferation
